# Asymmetric Influence of Vocalic Context on Mandarin Sibilants: Evidence From ERP Studies

**DOI:** 10.3389/fnhum.2021.617318

**Published:** 2021-04-22

**Authors:** Yaxuan Meng, Sandra Kotzor, Chenzi Xu, Hilary S. Z. Wynne, Aditi Lahiri

**Affiliations:** ^1^Faculty of Linguistics, Philology and Phonetics, University of Oxford, Oxford, United Kingdom; ^2^School of Education, Oxford Brookes University, Oxford, United Kingdom

**Keywords:** LDN, MMN, tongue height, vowel, mandarin sibilant

## Abstract

In the present study, we examine the interactive effect of vowels on Mandarin fricative sibilants using a passive oddball paradigm to determine whether the HEIGHT features of vowels can spread on the surface and influence preceding consonants with unspecified features. The stimuli are two pairs of Mandarin words ([sa] ∼ [ʂa] and [su] ∼ [ʂu]) contrasting in vowel HEIGHT ([LOW] vs. [HIGH]). Each word in the same pair was presented both as standard and deviant, resulting in four conditions (/standard/_[deviant]_: /sa/_[ʂa]_ ∼ /ʂa/_[sa]_ and /su/_[ʂu]_ ∼ /ʂu/_[su]_). In line with the Featurally Underspecified Lexicon (FUL) model, asymmetric patterns of processing were found in the [su] ∼ [ʂu] word pair where both the MMN (mismatch negativity) and LDN (late discriminative negativity) components were more negative in /su/_[ʂu]_ (mismatch) than in /ʂu/_[su]_ (no mismatch), suggesting the spreading of the feature [HIGH] from the vowel [u] to [ʂ] on the surface. In the [sa] ∼ [ʂa] pair, however, symmetric negativities (for both MMN and LDN) were observed as there is no conflict between the surface feature [LOW] from [a] to [ʂ] and the underlying specified feature [LOW] of [s]. These results confirm that not all features are fully specified in the mental lexicon: features of vowels can spread on the surface and influence surrounding unspecified segments.

## Introduction

To comprehend spoken language, listeners need to decode the incoming speech stream and segment it into units which map onto the phonological representations of words. However, the incoming acoustic cues for consonants and vowels can vary quite substantially due to factors such as context, speaking rate, and speaker characteristics. Nevertheless, mature listeners rarely experience any difficulty in recognizing spoken words and inferring the intended message (Marslen-Wilson, 1984; Norris et al., 1995; Lahiri and Reetz, 2002, 2010).

The speech signal varies in different contexts where the realization of a particular sound can differ within and across individual words (cf. Holt and Kluender, 2000). Furthermore, contextual modifications (contiguous sounds affecting each other such as vowels affecting consonants, consonants affecting other consonants, etc.) can alter the pronunciation of a sound quite drastically. A familiar example is that of place assimilation where the underlined medial sequences [ng] in *greengage* or [np] *gunpoint* are habitually articulated as [ŋg] and [mp] respectively. Here, the place of articulation of the [CORONAL] nasal [n] is affected by that of the following consonant, transforming it into a [DORSAL] [ŋ] or [LABIAL] [m] nasal. Vowels can also affect consonants as is seen in word pairs such as *face* ∼ *facial* or *commerce* ∼ *commercial*, where the final sound [s] of the first word of each pair becomes [ʃ] in the context of the vowel [i] when suffixed with -*ial* [iəl]. Here the [i] is no longer pronounced; however, in other contexts, such as in *dictator* ∼ *dictatorial*, the vowel [i] does not change. In this paper, we investigate brain responses to variability in sound sequences where vowels alter neighboring consonants.

**FIGURE 1:**
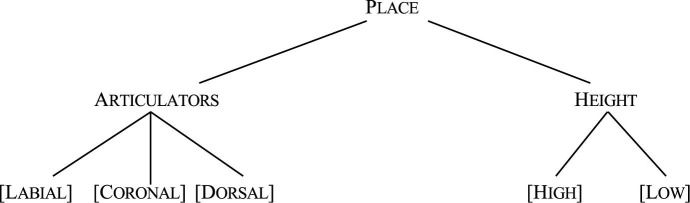
Feature organization of PLACE OF ARTICULATION in FUL.

The effect of one sound on another tends not to be symmetric. For example, in the example given above (*greengage* and *gunpoint*), the assimilation of the place of articulation is asymmetric. Although [CORONAL] [n] can change to [m] and [ŋ], the reverse is usually not the case: a [DORSAL] nasal, as in the sequence [ad] *kingdom* does not become ^∗^[nd] nor does the [LABIAL] nasal in *sometime* change to ^∗^[nt]. Thus, [CORONAL] consonants such as [n] can assimilate easily to the place of articulation of the following [LABIAL] (e.g., [p], [b]) or [DORSAL] consonants (e.g., [k], [g]) but not vice versa (cf. Cornell et al., 2013). One approach to capture this asymmetry is to assume that not all features or properties of consonants and vowels are fully specified in the lexicon (cf. the Featurally Underspecified Lexicon (FUL) model; Lahiri and Reetz, 2010; Scharinger et al., 2012; de Jonge and Boersma, 2015; Schluter et al., 2016; Højlund et al., 2019; Kotzor et al., 2020). In this model, consonants and vowels are defined by PLACE OF ARTICULATION which include ARTICULATOR features such as [CORONAL], [DORSAL], [LABIAL], and HEIGHT features [HIGH] and [LOW]. Of these, [CORONAL] is assumed to be universally underspecified (see Figure 1).

Since each word has a unique phonological representation, the features extracted from the acoustic signal are used to map speech onto underlying representations. Listeners process the variable speech signal and parse it into features which are then directly mapped onto the lexicon (Lahiri and Reetz, 2010; Kotzor et al., 2020). This mapping from the features in the signal to the lexicon is based on a ternary logic*: match, mismatch*, *and no-mismatch*. The first two options are transparent: *match* equates to the feature from the signal matching the lexicon completely while *mismatch* occurs when there is a conflict. Thus, the feature [CORONAL] from the acoustic signal of [n], for instance, will *mismatch* with the lexically represented feature [LABIAL] of [m]. The *no-mismatch* condition suggests a level of tolerance and is particularly important for underspecified features such as [CORONAL]. Consequently, [LABIAL] extracted from the signal of [m] will be in a no-mismatch relationship with [n] since its place feature [CORONAL] is not specified. Thus, during speech processing, all words in the lexicon with matching and no mismatching features are activated, but when mismatching features are encountered, words are deactivated.

There has been considerable evidence from both behavioral and neurophysiological studies for the underspecification of [CORONAL] place of articulation (Lahiri and Reetz, 2002, 2010; Eulitz and Lahiri, 2004; Cornell et al., 2011). For instance, the mismatch negativity (MMN) component has been used widely as a robust measure to examine [CORONAL] underspecification (Eulitz and Lahiri, 2004; Cornell et al., 2011). The MMN component, which usually peaks at 100–250 ms after the onset of a stimulus, signals the automatic or pre-attentive detection of an infrequent change in regular auditory stimulations (Näätänen et al., 2007). The MMN can be elicited by the deviant that violates the representation of repetitive standards before the occurrence of that deviant, suggesting that the sensory memory trace of preceding stimuli is compared against incoming sounds (Näätänen and Winkler, 1999; Horváth et al., 2008). The amplitude or latency of the MMN component depends on the magnitude of the stimulus deviation, with larger deviance resulting in an increase in amplitude and shorter latencies (Näätänen et al., 2007). In MMN studies examining coronal underspecification (e.g., Eulitz and Lahiri, 2004; Roberts et al., 2014), [CORONAL] deviants elicit larger MMN amplitudes in the context of, for instance, [LABIAL] standards as this creates a mismatch while deviants which are fully specified (e.g., [LABIAL]) and occur in the context of a [CORONAL] standard result in an attenuated MMN amplitude (no-mismatch).

Similar arguments have been made for ARTICULATOR features for vowels. Asymmetric MMN contrasts also support the concept of underspecified representations for vowels. Cornell et al. (2011) compared the phonological representations of vowels [ø] and [o] in the mental lexicon by means of MMN. The vowel [ø] is [CORONAL] and thus underspecified for its place of articulation while the vowel [o] is specified as [DORSAL]. In the context of a series of standard [ø], a fully specified phonetic [o] is a less different stimulus (i.e., no mismatch) than a deviant [ø] in the context of a series of fully specified standard [o] (i.e., a mismatch). Asymmetry occurs such that a deviant [o] in a standard [ø] context ([o]_/__ø__/_) elicits a smaller MMN than a deviant [ø] in an [o] context ([ø]_/o/_). Here, the representation activated by the repeated processing of standard stimuli is from a long-term memory trace, and associated to the underlying representation in the mental lexicon. In contrast, the sound percept elicited by the deviant stimulus corresponds to the surface representation, which is formed by the phonological features extracted from the acoustic signal (Eulitz and Lahiri, 2004; Cornell et al., 2011). The change detection response reflects the contrast between the underlying and surface representations.

Comparing both ARTICULATOR and TONGUE HEIGHT features, Kotzor et al. (2020) examined asymmetric ARTICULATOR features as well as symmetric HEIGHT features in vowels in words and non-words (Table 1). They contrasted the ARTICULATOR asymmetry in the vowels [ɛ] [CORONAL] and [ɔ] [DORSAL] in the verbs *get* [gɛt] ∼ *got* [gɔt] and the pseudowords *^∗^gef* ∼ *^∗^gof.* In the same study, conflicting fully specified, and hence symmetric, HEIGHT features which mismatch in both directions were also compared while keeping the ARTICULATOR feature [CORONAL] constant: *sit* [sIt] ∼ *sat* [sæt] and *^∗^sif* ∼ *^∗^saf*, where [I] [HIGH] and [æ] [LOW] conflict and hence mismatch. While the place features [CORONAL] and [DORSAL] were predicted to elicit asymmetric MMNs in both words and pseudowords, the height features, which are both fully specified, mismatch and should thus elicit high MMNs of comparable amplitude regardless of which vowel occurs as standard or deviant. The results confirmed their hypotheses: due to [CORONAL] underspecification, [CORONAL] and [DORSAL] place features elicited asymmetric MMNs, while conflicting height features [HIGH] and [LOW] mismatched and the MMNs did not differ.

**TABLE 1:**
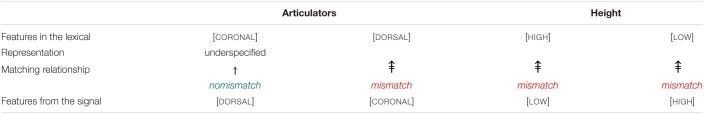
The mismatching and matching relationships in the study by Kotzor et al. (2020).

*The symbol ↑ represents nomismatch and ⇞ represents mismatch.*

So far, we have discussed pairs of individual features on which the influence from surrounding segments have been kept constant. However, in normal speech, contiguous consonants, and vowels always lead to coarticulation or spreading of features, some more than others. Thus, in a VOWEL + NASAL sequence such as the English word *an*, the [NASAL] feature of [n] can spread to the vowel [ae] leading to [æ̃n]. This assimilation also has processing consequences (Lahiri and Marslen-Wilson, 1991). In English, this is purely allophonic, which means that the nasalization is entirely predictable and there is no real phonemic contrast between oral and nasal vowels; e.g., *cad* [kæ̃aed] vs. *can* [k^h^æ̃n]. Nevertheless, on perceiving nasality on the vowel, English listeners can anticipate a following nasal consonant. e.g., [n] can be anticipated after hearing the sequence [k^h^æ̃] in *can*. In this paper, we address the consequence of similarities and differences of more complex CV units where the feature ARTICULATOR is kept constant, but HEIGHT spreads from vowels to consonants. Using an MMN paradigm, we examine the contrastive [CORONAL, STRIDENT] consonants [s] and [ʂ] which differ in HEIGHT in the context of both [HIGH] and [LOW] vowels. The relevant vowels are [u] and [a] which also differ in HEIGHT: [sa]∼[ʂa] and [su]∼[ʂu] (as shown in Table 2). At first glance, the pairs appear to be straightforward; however, the underlying phonological representation of the features for these pairs depends not only on the phonemes but on the general phonological inventory of Mandarin.

**TABLE 2:**
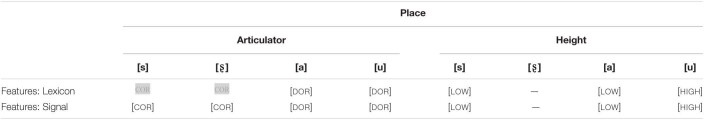
Critical features for the Mandarin CV sequences.

*The feature [CORONAL] in the representation is underspecified and is shown in gray.*

The phonological feature specifications within a language are determined by the number of contrastive segments. In Mandarin, there are two sets of [CORONAL] obstruents: *dental* [t, t^h^, ʦ, ʦ^h^, s] and *retroflex* [tʂ, tʂ^h^, ʂ]. There are fewer retroflex consonants than dentals in Mandarin: Duanmu (2007) states that the retroflex series is a “major characteristic of Standard Chinese (SC) speakers from Beijing” (p. 24) and that speakers of other Chinese dialects replace the retroflex with the dental; e.g., there would be no distinctions between [sa] “sprinkle” and [ʂa] “stupid.” To distinguish between the two types of [CORONAL] obstruents, our feature system uses the HEIGHT features [HIGH] and [LOW] (cf. Lahiri, 2018). Based on their acoustic characteristics, the retroflex consonants would be characterized as [HIGH] and the dentals as [LOW]: dental sibilants have more energy in the higher frequencies compared to retroflexes and palatals (Stevens and Blumstein, 1975; Lahiri and Reetz, 1999; Lahiri and Kennard, 2019; Kennard and Lahiri, 2020). However, Mandarin only has a two-way contrast in the voiceless sibilant fricatives [s] and [ʂ]; thus, it is only necessary to lexically specify one of these phonemes; the other remains *unspecified*. Since there are more dental consonants than retroflexes and since the dentals are less likely to vary in comparison to the retroflexes, the dentals would be more likely to be specified for HEIGHT in the lexicon.

Further evidence of the specification of the HEIGHT feature is provided by the co-occurrence restriction that certain adjacent identical elements are prohibited in consonant-glide sequences (Yip, 1988; Wiese, 1997; Duanmu, 2007). As for vowels, descriptively Mandarin allows five basic vowels where [i u y] are high vowels, [ə] is a mid vowel and [a] is usually characterized as a low vowel. In terms of features, the mid vowel is underspecified while the high and the low vowels are specified. Mandarin syllables can only have single consonants as onsets and codas and no clusters are permitted. Thus, since all initial consonants followed by high vowels /i u y/ attain a secondary articulation described as glide spreading, /CuC/ becomes [C^*w*^uC] where the [C^*G*^] holds a single position in the onset. As we can see, [s] can occur with both high and non-high vowels, such as /suuŋ4/ [s^*w*^uŋ] 送 “send” or /suən1/ [s^*w*^ən] 孙 “grandchild,” where the glide formation rule turns the vowel [u] into a glide leading to a secondary articulation [s^*w*^]. Since [s] is specified as [LOW] and the glides are high, the secondary articulation is allowed. Had/ʂ/ been specified for [HIGH], the sequence /ʂuu/> [ʂ^*w*^u] “to lose” would not have been permitted because of identical height features (Table 2). Crucially, the feature [HIGH] is not specified in the language for any of the consonants, thus allowing them to take on the secondary articulations triggered by high vowels. We will examine the two sibilants [s] and [ʂ], which are differentiated in HEIGHT, in combination with two vowels also differing in HEIGHT: [u] and [a].

As we mentioned above, not only do ARTICULATOR features such as [LABIAL] or [DORSAL] spread leading to assimilation in words such as *greengage*, but TONGUE HEIGHT features such as [LOW] or [HIGH] can also spread to preceding unspecified segments (Kunisaki and Fujisaki, 1977; Mann and Repp, 1980; Lahiri and Reetz, 2002). In a study by Kunisaki and Fujisaki (1977), a continuum of synthetic fricative sounds varying from [ʃ] to [s] was combined with different vowels. The category boundary was found to shift to [s] when followed by vowels [u] or [o], while it shifted toward [ʃ] in the context of [a] or [e], suggesting an effect of vocalic context on fricative consonant perception. Thus, it appears that simple coarticulation in contiguous segments can influence perception. Similarly, Mann and Repp (1980) also found that listeners were more likely to perceive a synthetic fricative consonant from a [ʃ]∼[s] continuum as a [s] in the context of [u] compared to the context of [a]. The authors attributed the influence of vowel on consonant to an assimilatory change where the vowel rounding and consonant place of articulation coarticulated (Mann and Soli, 1991). If this is the case, symmetric MMNs between the phonological contrasts [sa] ∼ [ʂa] and [su] ∼ [ʂu] would be expected independent of the direction of presentation of the standard and deviant, as no feature is unspecified.

In contrast, asymmetric MMNs would be expected in the reversal of phonological contrasts if the influence of vocalic context is due to the spreading of features on the surface. Given that certain features are underspecified, the influence of vocalic context will be greater if the contiguous segment lacks a feature. The fricatives [s] and [ʂ] share the same ARTICULATOR feature [CORONAL] as well as the MANNER feature [STRIDENT], but differ with respect to their HEIGHT features (see Table 3). As only [LOW] is specified, the HEIGHT features of following vowels could spread to preceding [ʂ] but not [s]. In other words, the surface height feature of [ʂ] is determined by the following vowels. In their underlying representations, dental [s] is assumed to be [LOW] with [CORONAL] underspecified, while retroflex [ʂ] is assumed to be underspecified for ARTICULATOR and unspecified for the HEIGHT feature. Since [ʂ] lacks specification of TONGUE HEIGHT features, it can take on the HEIGHT features of the following vowel [u] and [a] (Table 3 a, b). In contrast, [s] is specified for [LOW], and thus, phonologically, it is not affected by the features of the following vowel and retains its own HEIGHT feature (Table 3 c, d). If this feature spreading account holds, then we would assume that, although the vowels are identical and both sibilant fricatives are [CORONAL], [sa] vs. [ʂa] and [su] vs. [ʂu] should elicit different activation patterns: specifically, we predict that [sa]∼[ʂa] will lead to symmetric activation while [su]∼[ʂu] will not.

**TABLE 3:**
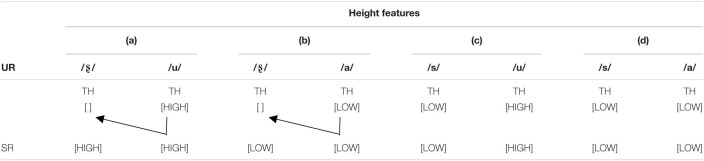
The spreading of HEIGHT features from vowel to consonant.

Along with MMN, an additional negativity, the late discriminative negativity (LDN), was observed in our study. The LDN is a recently established component found in oddball paradigms and serves as an index of phonological discriminative abilities (Hill et al., 2004; Horváth et al., 2009; Jakoby et al., 2011; David et al., 2020). Similar to the MMN, the LDN is also an automatic response associated with higher cognitive processes and may represent the recruitment of additional cortical resources needed to extract the phonological differences between the standard and deviant stimulus and form phonological representations (Shestakova et al., 2003; Hill et al., 2004; Zachau et al., 2005; Barry et al., 2009). The LDN can be elicited by both speech and non-speech sounds, and its amplitude was found to be related to the difficulty in discriminating the stimuli (Korpilahti et al., 1995, 2001; Schulte-Körne et al., 1998). For example, Yu et al. (2017) compared the processing of Mandarin disyllabic non-words with different inter-stimulus intervals (ISIs) between Mandarin- and English-speaking groups. For both groups, robust MMNs to contrasts with either similar or contrastive lexical tones at shorter ISIs were observed. Compared to the English group, a larger LDN was only found for the Mandarin group when processing contrasts at longer ISIs, especially those with similar lexical tone. These results suggest that it is easier to discriminate the acoustic correlates of lexical tone at shorter ISIs. To discriminate words at longer ISIs, language-specific experience is necessary. Following the FUL model, Hestvik and Durvasula (2016) examined the underspecification-driven asymmetry in the processing of the English contrast between /d/, which is underspecified for [VOICE], and /t/, which is specified for the feature [SPREAD], using the oddball paradigm. The LDN component exhibits the same asymmetry as the MMN with a mismatch for /t/_[d]_ but not for /d/_[t]_. Based on these previous studies, we anticipate that, consistent with the activation patterns of MMN, [sa]∼[ʂa] will lead to symmetric LDNs for the subtle difference between standard and deviant, while [su]∼[ʂu] will not.

### Methodology

The presented study examines the interactive effect of vowels on fricative sibilants to determine whether the TONGUE HEIGHT features of vowels can spread on the surface and influence unspecified preceding consonants. Coarticulation, which leads to feature spreading, would suggest symmetric MMNs between phonological contrasts, independent of the direction of presentation of the standard and deviant if the features are fully specified in both standards and deviants. In contrast, asymmetric MMNs would be expected between the two directions of presentation (i.e., standard vs. deviant) of phonological contrasts where the HEIGHT feature [HIGH] is unspecified in one of the stimuli.

Since Mandarin also has a tonal contrast, it was necessary to keep the tones consistent across the stimuli. Two monosyllabic word pairs with Tone 1, [sa]∼[ʂa] and [su]∼[ʂu], were used as the standard and deviant stimuli. Mandarin is a language where one syllable corresponds to one morpheme in most cases, with each syllable being comprised of an optional initial consonant, optional glide, a vowel, and an optional final consonant [*n* (n) *or ng* (ŋ)]. We already described the two voiceless sibilant fricatives in Standard Chinese (SC, or Mandarin), represented as the dental/alveolar [s] and retroflex [ʂ]. Here, the retroflex [ʂ] in Mandarin is different from the palatoalveolar [ʃ] in English in terms of the consonant position and air flow through the mouth. The palatoalveolar is pronounced with the air flow through the tongue blade and even a portion of the front part of the tongue. For the retroflex, the air flow is more limited to the tongue tip/blade region (Lin, 2007). Here, we follow Duanmu’s (2007) position and treat the voiceless fricative sibilants in Mandarin as a two-way contrast: the dental/alveolar [s] and retroflex [ʂ]. Unlike the two-way contrast between fricative sibilants, the vowels in Mandarin are categorized into a three-way height distinction, including three high vowels [i y u], one mid vowel [ə], and one low vowel [a] (Duanmu, 2007). As mentioned above, both [HIGH] and [LOW] should be specified when there is a three-way height difference (Lahiri and Reetz, 2010). Therefore, the surface and underlying representations of [a] and [u] are consistent in that [a] is assumed as [LOW][DORSAL] and [u] is assumed as [HIGH][DORSAL], respectively (refer to Table 2).

**FIGURE 2:**
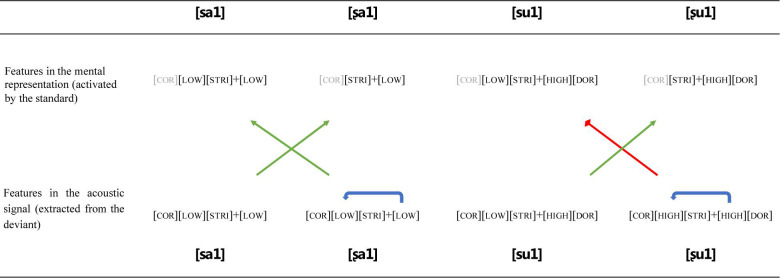
Predictions made about the feature conflict in the four experimental conditions. The arrows illustrate the main statistical model. The blue arrow reflects the feature spread on the surface from vowel to consonant. The green arrows indicate combinations of standard and deviant stimuli in no-mismatch conditions and the red arrow represents the mismatch condition.

We predict that: (1) for the [sa]∼[ʂa] word pair, no conflict will occur between /sa/_[ʂa]_ and /ʂa/_[sa]_ (/Standard/_[__Deviant__]_). As discussed above, features spread on the surface with the HEIGHT feature of the vowel affecting the preceding unspecified sibilant. Therefore, the HEIGHT feature [LOW] of the vowel [a] spreads to [ʂ] when [ʂa] occurs as a deviant. As a result, the surface HEIGHT features of [ʂa] in the /sa/_[ʂa]_ condition become [LOW] + [LOW] with the [CORONAL] PLACE feature being the only no-mismatching feature. For the /ʂa/_[sa]_ condition, both the PLACE and HEIGHT features are in a no-mismatch relationship with the underlying representations and hence no-mismatching patterns are found. Therefore, symmetric MMNs and LDNs are predicted for these two conditions (as shown in Figure 2).

Our second prediction (2) is that, for the [su]∼[ʂu] word pair, a phonological conflict occurs between /su/_[ʂu]_ and /ʂu/_[su]_, causing the deviant [ʂu] in a standard [su] context to elicit a larger MMN and LDN than a deviant [su] in a [ʂu] context. For the /su/_[ʂu]_ condition, the HEIGHT feature spreads from [u] to [ʂ], resulting in the surface HEIGHT features [HIGH] + [HIGH] of [ʂu]. The HEIGHT feature of [s], however, is specified as [LOW] resulting in the underlying HEIGHT features [LOW] + [HIGH] for [su]. Thus, a mismatch occurs in the /su/_[ʂu]_ condition. Compared to /su/_[ʂu]_, there is no surface feature spread in the /ʂu/_[su]_ condition so that the HEIGHT and PLACE features of deviant [su] no mismatch the underlying representation of standard [ʂu]. Consequently, asymmetric MMNs and LDNs are predicted with a larger amplitude for /su/_[ʂu]_ than /ʂu/_[su]_.

If, on the other hand, we assume a phonemic representation with every feature fully specified in all sounds, all variants should mismatch to the same degree, as the spreading from [u] would not alter the specification of [LOW] in [ʂ]. In such a case, we would expect to see symmetric MMN and LDN responses for both pairs of words, regardless of the direction of presentation (i.e., which is the standard and which the deviant).

## Materials and Methods

### Participants

Twenty-one students (11F/10M, mean age = 23.86 years), recruited at the University of Oxford, participated in the study. They were all native Mandarin speakers who lived in China until adulthood and were residing in Oxford at the time of testing. All participants had normal or corrected-to-normal vision and self-reported as right-handed (a modified version of the Edinburgh Handedness Inventory was also used to assess handedness, Oldfield, 1971). No history of neurological disorders or hearing deficits was reported. The study was approved by the Central University Research Ethics Committee (CUREC) and written informed consent was acquired from subjects prior to the experiment. They were compensated for their participation.

### Stimuli

Two pairs of Mandarin monosyllabic words that differ only in initial fricative consonants were used in the experiments ([sa] ∼ [ʂa]; [su] ∼ [ʂu]). Monosyllabic words are plentiful in Chinese and thus are polysemous. Each permissible syllable, with any one of the four lexical tones, could represent various meanings. As there are only two fricative sibilants in Mandarin, it is difficult to construct pseudowords with a combination of vowels differing in TONGUE HEIGHT. Thus, our four stimuli are all words, each of which predictably has several meanings. The most obvious meanings of the four syllables are as follow: [sa] 撒 “let go”∼ [ʂa] 沙 “sand”; [su] 苏 “place name”∼ [ʂu] 输 “to lose.” According to the SUBTLEX-CH, the frequencies for these syllables are 3.46 ([sa]^1^), 3.34 ([ʂa]^1^), 3.17 ([su]^1^), and 3.35 ([ʂu]^1^) (Cai and Brysbaert, 2010).

**FIGURE 3:**
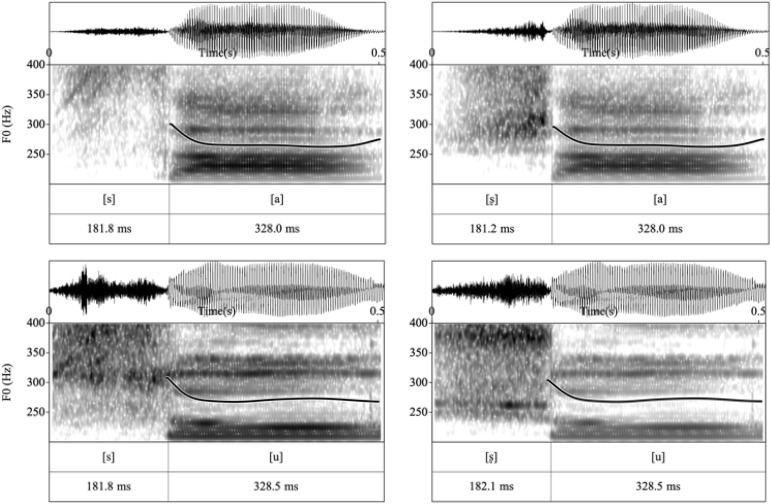
Oscillograms (above), spectrograms (below, 0–7,000 Hz), and F0 tracks of the four stimuli. All the stimuli are Tone 1 syllables.

As expected, the spectrogram of the same fricative varies depending on the following vowel (Figure 3). The coarticulation was maintained in order to preserve the naturalness of the stimuli. Each pair contained contrasting coronal fricatives [s] and [ʂ] embedded in respective vowel contexts: the [a] with feature [LOW], and the [u] with features [HIGH] and [DORSAL]. Since Mandarin has a lexical contrast in tones, it was important to control for this as well. All syllables were chosen to have lexical Tone 1, which is usually described as a high-level tone (Duanmu, 2007). Thus, the pitch is held at a constant level.

Multiple repetitions of four syllables were recorded by a female native speaker of Mandarin in a sound-attenuated recording room using a professional quality USB microphone (Røde NT-USB) at a sampling rate of 44.1 kHz. From these syllables, we generated four naturally sounding stimuli recordings. A representative utterance of each syllable with similar duration was selected. The recordings were extracted and segmented using the speech analysis program Praat (Boersma and Weenink, 2018). The [a] and [u] vowels in [sa] and [su] were cross-spliced to the corresponding [ʂ] consonant in each pair such that the acoustic differences between the stimuli in each pair were minimized to the contrasting consonants. As shown in Figure 3, the vowel portions in each pair were identical.

Across pairs, stimuli were also controlled for duration (Figure 3). In the recordings, the vowel [u] was slightly longer than [a], so some trailing pulses at the end of [u] were removed. Likewise, some initial pulses of noise were removed in [su] and [ʂu] because their frication duration was slightly longer than those of [sa] and [ʂa]. Such manipulations avoided the parts of formant transitions in order to minimize the distortions of F0 and spectral features. Therefore, all initial consonants were approximately 182 ms, the vowels 328 ms, and the overall duration of a syllable was about 510 ms (as shown in Figure 3). The intensities of all stimuli were equalized in Praat.

### Experimental Procedure

Two pairs of words with Tone 1 were presented to participants during the experiment. Each word pair was presented in two conditions; one with a [s] consonant as the deviant and a [ʂ] consonant as the standard, and one with the direction of presentation reversed (see Table 4). The word pairs will be described respectively as /sa/_[ʂa]_ ∼ /ʂa/_[sa]_ and /su/_[ʂu]_ ∼ /ʂu/_[su]_ in the paragraphs below (/standard/_[__deviant__]_).

**TABLE 4:** Task design in MMN tasks.

	Experiment 1		Experiment 2	
	Standard	Deviant	Standard	Deviant
Block 1	[sa]	[ʂa]	[su]	[ʂu]
Block 2	[ʂa]	[sa]	[ʂu]	[su]

As a result of this reversed design, four oddball blocks were presented to each participant with the sequence of blocks counterbalanced among the participants. Within each block, the deviant occurred pseudo-randomly among the standards with a probability of 15%. Any two adjacent deviants were separated by at least two standards. A total of 610 stimuli, with ten continuous standard stimuli occurring at the beginning, were presented in each block. To eliminate the influence of a rhythmic pattern established by temporal characteristics of the acoustic stimuli, the ISI between standard and deviant varied randomly between 350 and 650 ms.

### EEG Recordings

EEG recordings were made using a Biosemi ActiveTwo amplifier with 64 sintered Ag/AgCl pin electrodes placed in a 10–20 montage, online referenced to the mastoids. EOG activity was measured using four facial electrodes (IO1, IO2, LO1, and LO2). All electrode offsets (in an active-electrode system this is comparable to impedance) were kept below 30 mV and signals were sampled at 2,048 Hz. The audio stimuli were presented through headphones and participants watched a self-selected silent documentary during the experiment. All subjects participated in all four blocks and the order of the four blocks was counterbalanced across subjects. The total duration of the experiment was about 90 min and subjects had a short break between blocks.

### Data Analysis

EEG data were analyzed offline using EEGLAB 14.1.2b. All continuous data were digitally-filtered offline in 0.3–30 Hz range using a finite impulse response filter (FIR filter). Bad channels and artifacts were detected and removed automatically by the artifact subspace reconstruction (ASR) method as implemented in the Clean Raw Data plug-in. EEG data were re-referenced to the linked mastoids for all analyses except for mastoid amplitudes. Using an independent components analysis (ICA, Delorme and Makeig, 2004), ICA components that may represent eye blinking, lateral eye movement, muscle activity, or channel noise were detected and excluded from further analysis. Furthermore, epochs were created from −100 to 800 ms with the time windows from −100 to 0 ms used as a baseline. An additional artificial detection was carried out so that trials were rejected if they exceeded an amplitude of 100 μV. In addition, any participant with an acceptance rate lower than 70% was excluded, which led to the exclusion of three participants from further analysis. Finally, the first ten responses of each block and two standards after each deviant were rejected in the grand average. For the difference waves, a deviant-minus-standard calculation was carried out for each participant and condition; namely, the difference was generated by subtracting the waveform of the stimuli when it was presented as standard in one block from that of the same stimuli when it was presented as deviant in another block.

## Results

Based on visual inspection of the grand-average waveform, the amplitudes of MMN and LDN were determined for each participant and condition as the mean amplitude within 140–180 ms and 320–360 ms after the onset of stimuli at *Fz.* According to previous studies, both the MMN and LDN are typically maximal over fronto-central electrode sites (Näätänen et al., 1992; Jakoby et al., 2011). Thus, the analyses were restricted to twelve frontocentral electrodes (*AF3, AFz, AF4, F3, Fz, F4, FC3, FCz, F4, C3, Cz*, and *C4*). For each experiment, repeated ANOVAs with *Condition*, *Vowel*, *Laterality* (left, middle, and right), and *Gradient* (AF-, F-, FC-, and C- line) as within-subject variables were carried out for mean amplitude and peak latency, respectively. For all analyses, degrees of freedom were adjusted according to the method of Greenhouse–Geisser.

### Mismatch Negativity

**FIGURE 4:**
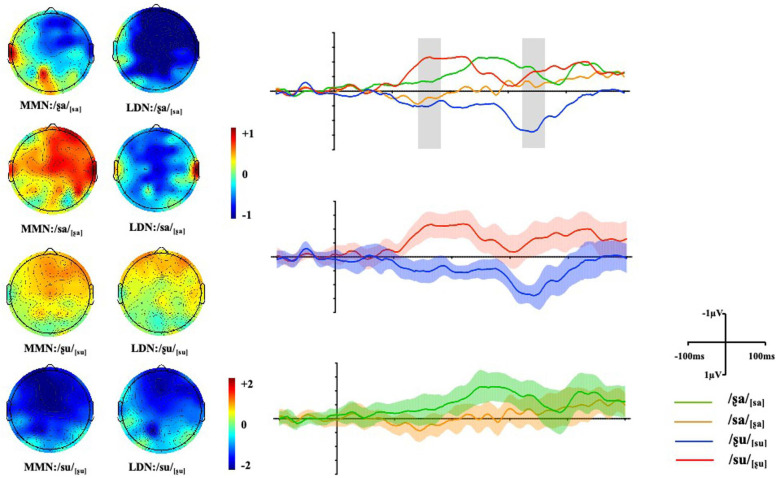
Maps display the topographic distribution of the mean amplitude in both the MMN and LDN analysis windows from 140–180 ms and 320–360 ms respectively. Grand-average difference waveforms of all four conditions at *Fz* (see Supplementary Material for the waveforms at all selected electrodes). Shade areas show 95% confidence intervals.

Repeated ANOVAs were conducted and significant main effects of *Condition* and *Vowel* were found, *F*_1_(1, 17) = 6.99, *p*_1_ = 0.017, η*_*p*_^2^* = 0.29; *F*_2_(1, 17) = 5.62, *p*_2_ = 0.030, η*_*p*_^2^* = 0.25. However, the interaction between *Vowel* and *Condition* was also significant, *F*(1, 17) = 21.39, *p* < 0.001, η*_*p*_^2^* = 0.56. *Post hoc* analyses were conducted and the results showed that for vowel [a], there was no significant difference between the mean amplitude of /ʂa/_[sa]_ and /sa/_[ʂa]_, *F*(1, 17) = 2.69, *p* = 1.12, η*_*p*_^2^* = 0.14, indicating non-significant difference in MMN amplitudes between the features of the /ʂa/_[sa]_ and /sa/_[ʂa]_ word pairs as in both pairs the feature [CORONAL] of the deviant generates a no-mismatch with the underspecified [CORONAL] feature of the standard (as shown in Figure 4). Compared to the /sa/_[ʂa]_ pair, the surface feature [LOW] of the consonant [s] in /ʂa/_[sa]_ is also in a no-mismatch relationship with the underlying unspecified TONGUE FEATURE of [ʂ] and therefore, the mean amplitude of the /ʂa/_[sa]_ pair was more negative than that for /sa/_[ʂa]_ (Figure 4). However, this difference did not reach statistical significance. For word pairs with vowel [u], the amplitude of the MMN response triggered by /su/_[ʂu]_ was significantly more negative than that for /ʂu/_[su]_, *F*(1, 17) = 33.84, *p* < 0.001, η*_*p*_^2^* = 0.67. As predicted by the FUL model, the asymmetric HEIGHT pair shows a larger MMN in the mismatch condition, when the HEIGHT feature [HIGH] of the deviant [ʂ] maps onto the pre-activated HEIGHT feature [LOW] of the standard [s]. A reduced MMN amplitude was found in the reversed condition /ʂu/_[su]_, where the features [LOW] [CORONAL] of deviant [s] are in a no-mismatch relationship with the underlying features of standard [ʂu]. Furthermore, the mean amplitudes of conditions where the initial consonant of deviants was [s], were more negative when combining with vowel [a] than with [u], *F*(1, 17) = 5.71, *p* = 0.029, η*_*p*_^2^* = 0.25. For conditions where the initial consonant of the deviants was [ʂ], the amplitude was more negative when followed by vowel [u] than vowel [a], *F*(1, 17) = 23.90, *p* < 0.001, η*_*p*_^2^* = 0.58.

**FIGURE 5:**
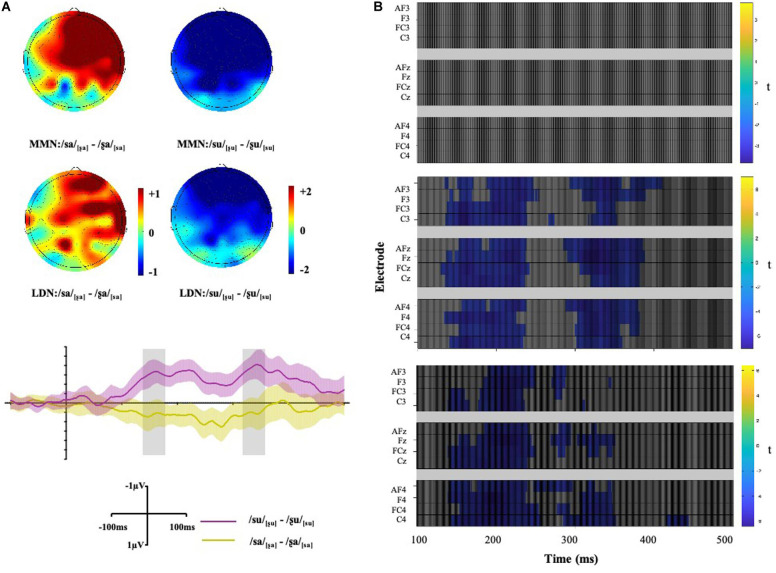
**(A)** Maps display the topographic distribution of the mean amplitude difference between conditions in both the MMN (140–180 ms) and LDN (320–360 ms) analysis windows. Grand-average difference waveforms between conditions at *Fz*. Shade areas show 95% confidence intervals. **(B)** Within-subject *t*-tests between conditions at all selected electrodes after multiple comparison corrections using mass univariate ERP toolbox (Groppe et al., 2011). The difference is represented at each time point from 100 to 500 ms relative to the stimulus onset. Difference between /sa/_[ʂa]_ ∼ /ʂa/_[sa]_ (top); difference between /su/_[ʂu]_ ∼ /ʂu/_[su]_ (middle); difference between (/su/_[ʂu]_ - /ʂu/_[su]_) and (/sa/_[ʂa]_ - /ʂa/_[sa]_) (bottom).

To further investigate these patterns of activation in both directions when followed by different vowels, the wave difference between /su/_[ʂu]_ and /ʂu/_[su]_ was compared to that between /ʂa/_[sa]_ and /sa/_[ʂa]_ within the 140–180 ms time window. The results showed significant differences across all gradients, *ps* < 0.001, suggesting asymmetric pattern of activation (see Figure 5).

### Late Discriminative Negativity

Repeated ANOVAs were conducted for the LDN component and a three-way interaction between *Vowel*, *Condition*, and *Gradient* was also significant [*F*(3, 51) = 7.32, *p* = 0.003, η*_*p*_^2^* = 0.30]. *Post hoc* analyses were conducted, and the results showed that no significant difference was found between the /ʂa/_[sa]_ and /sa/_[_ʂ_a]_ conditions across gradients. Non-significant LDNs were also observed when surface features no mismatch the underlying underspecified [CORONAL] or unspecified [HIGH]. For words with the vowel [u], a significant difference was found between /ʂu/_[su]_ and /su/_[ʂu]_ where the mean amplitude of /su/_[ʂu]_ was more negative than that of /ʂu/_[su]_ at *AF*-, *F*-, *FC-* and *C-* [*t*_1_ (17) = −6.24, *p*_1_ < 0.001, *hedge’s g_1_* = 2.02; *t*_2_ (17) = −6.69, *p*_2_ < 0.001, *hedge’s g_2_* = 2.15; *t*_3_ (17) = −6.34, *p*_3_ < 0.001, *hedge’s g_3_* = 2.21; *t*_4_ (17) = −5.75, *p*_4_ < 0.001, *hedge’s g_4_* = 1.89]. Therefore, the subtle difference between the [sa]∼[ʂa] word pair may suggest symmetric LDNs while the TONGUE HEIGHT difference in the [su]∼[ʂu] word pair elicits an asymmetric late negativity. In addition, the mean amplitudes of conditions where the initial consonant of the deviants was [s] were more negative when combined with the vowel [a] than with [u] at *AF*-, *F*-, *FC-* and *C-* [*t*_1_ (17) = −6.80, *p*_1_ < 0.001, *hedge’s g_1_* = 1.94; *t*_2_ (17) = −6.32, *p*_2_ < 0.001, *hedge’s g_2_* = 1.96; *t*_3_ (17) = −6.33, *p*_3_ < 0.001, *hedge’s g_3_* = 2.01; *t*_4_ (17) = −5.40, *p*_4_ < 0.001, *hedge’s g_4_* = 1.79]. The wave difference between /su/_[ʂu]_ and /ʂu/_[su]_ was further compared to that between /ʂa/_[sa]_ and /sa/_[ʂa]_ in the 320–360 ms time window. The results showed that the difference was significant across all gradients, *ps* < 0.01, suggesting asymmetric pattern of activation (see Figure 5).

## Discussion

The present study was designed to examine the interactive effect of different vowels on fricative sibilants. We compared both the MMN and LDN responses to two pairs of Mandarin words ([sa]∼[ʂa] and [su]∼[ʂu]). The two consonants [s] and [ʂ] share the same place of articulation [CORONAL] but differ in TONGUE HEIGHT. As only the feature [LOW] is specified, the underlying representation of the consonant [ʂ] is unspecified for TONGUE HEIGHT. The vowels [a] and [u] mismatch in HEIGHT with [a] specified as [LOW], while [u] is specified as [HIGH]. As features can spread on the surface, the HEIGHT feature of the unspecified consonant [ʂ] changes when combined with different vowels.

Our results support the predictions of the FUL model (Lahiri and Reetz, 2002, 2010), which proposes that phonological contrasts can either match, mismatch or stand in a no-mismatch relation depending on whether the individual phonological features are fully specified or underspecified in the underlying representation. Previous studies have argued that the influence of vocalic context on fricative sibilants is due to the coarticulation of vowel rounding and consonant place of articulation (Mann and Soli, 1991). However, phonemic coarticulation would predict symmetric MMNs between phonological contrasts, independent of the direction of presentation of the standard and deviant. Thus, only an underspecification account can explain the asymmetry found in our results, as the features of vowels spread on the surface and the unspecification of TONGUE HEIGHT in the consonant [ʂ] leads to an asymmetric pattern depending on which stimulus is presented as standard and which as deviant (Lahiri and Reetz, 2002, 2010). Symmetric MMNs and LDNs were found between the no-mismatched contrasts (/ʂa/_[sa]_ ∼ /sa/_[ʂa]_). The feature [LOW] of vowel [a] spreads to the consonant [ʂ] when the [ʂa] is presented as deviant, resulting in the only no-mismatched feature being the underspecified [CORONAL] in both cases. When [ʂa] played the role as the standard, both the features [LOW] and [CORONAL] of consonant [s] resulted in a no-mismatch with the underlying representation of [ʂ]. In contrast, an asymmetric pattern was observed in the [su]∼[ʂu] word pair with the [HIGH] vowel [u]. When combined with the unspecified consonant [ʂ] as the deviant, the feature [HIGH] of [ʂ] conflicted with the underlying specified feature [LOW] of [s] and resulted in larger amplitudes of both MMN and LDN. No conflict was found when the feature [LOW] of the deviant [s] was in a no-mismatch relationship with the underlying unspecified [ʂ]. Consequently, the MMN and LDN amplitudes were significantly greater for the /su/_[ʂu]_ pair than for /ʂu/_[su]_.

Similar results for both symmetric and asymmetric MMN patterns were also reported by previous studies when considering both PLACE and MANNER features of consonants. Cornell et al. (2013) compared the phonological representations of four consonants [g], [d], [n], and [z], the first two being [PLOSIVE] and the latter two [NASAL] and [STRIDENT] respectively. Furthermore, the place feature of the first consonant is [DORSAL], while the remaining three are all [CORONAL]. The consonants were embedded in a non-word VCV structure, resulting in the sequences [egi], [edi], [eni], and [ezi]. Based on the FUL model, the features [PLOSIVE] and [CORONAL] are underspecified, while the others are specified in the mental representation. Asymmetric MMNs were observed in the /g/_[d]_ condition as the [CORONAL] extracted from the deviant [d] conflicts with the specified feature [DORSAL] which has been activated by the standard /g/. In the reversed condition /d/_[g]_, a non-conflicting situation occurs as the feature [DORSAL] extracted from the deviant [g] is tolerated (no mismatch) due to the underspecified [CORONAL] of the standard /d/. Similarly, the feature [PLOSIVE] extracted from the deviant [d] conflicts with the underlying specified [NASAL] of the standard [n] in the /n/_[d]_ condition, while no conflict occurs in the reversed condition /d/_[n]_ as [d] is underspecified for manner of articulation ([PLOSIVE]). In contrast, symmetric MMNs were found between [n] ∼ [z] as the features [NASAL] and [STRIDENT] are both fully specified and thus conflict equally in both directions. The results support our findings: both unspecified TONGUE HEIGHT and underspecified MANNER features can trigger asymmetric MMNs in different directions when the PLACE feature of the two consonants is kept constant. The difference is that the underspecified MANNER feature itself can trigger asymmetry while unspecified TONGUE HEIGHT feature needs to absorb additional features from surrounding segments. Therefore, different patterns of activation were found when followed by different vowels.

However, unlike the underspecification of [CORONAL], the lack of specification of [HEIGHT] is not universally applicable to all languages. It is central to the FUL model that the phonological representation of each segment is feature-based and constrained by universal properties, as well as language specific requirements (Lahiri and Reetz, 2002, 2010). Among these features, some are opposing binary pairs, such as consonantal ∼ vocalic and sonorant ∼ obstruent. The members of each pair are conflicting: a consonantal segment, for instance, cannot be vocalic and vice versa. Other features, such as [HIGH] and [LOW], are mutually exclusive but not binary. In other words, a segment cannot be both [HIGH] and [LOW] but can be neither. As discussed earlier, the number of contrastive segments in a certain language determines the specification of phonological features. In Mandarin, there is only a two-way contrast of voiceless fricative sibilants: the dental/alveolar [s] and retroflex [ʂ]. Here, the HEIGHT feature [HIGH]∼[LOW] is the only distinction between the two consonants as both of them are [CORONAL]. However, it is not necessary to specify both consonants as the phoneme that is not [HIGH] can be automatically categorized as [LOW] (Lahiri and Kennard, 2019; Kennard and Lahiri, 2020). This rule cannot be applied to segments with a three-way contrast, for instance, the Mandarin vowels. Different from two-way contrasts, both features [HIGH] and [LOW] are specified for a three-way contrast. Thus, the feature [MID] does not need to be stored and can be determined as the consequence of a binary distinction between high vs. non-high and low vs. non-low (Scharinger and Lahiri, 2010). Therefore, the results found in our study might not hold in investigations of the spreading of TONGUE HEIGHT features in other languages with a different number of contrastive segments.

Since the initial logic of the experiment was built into the framework of FUL’s feature model and assumptions regarding the matching algorithm, we discussed the results in that context. However, aside from the FUL model, there are other models focusing on perception asymmetry, such as the Natural Referent Vowel (NRV) framework (Polka and Bohn, 2003, 2011) and the Native Language Magnet (NLM) theory (Kuhl, 1991, 1992, 1993). In the NRV model, Polka and Bohn suggested that vowel perception is asymmetric with respect to the location of each vowel within a traditional articulatory or F1/F2 acoustic vowel space; namely, a change from a central vowel to a peripheral vowel (e.g., from [y] to [u]) would be much easier to discriminate than the same change in the reverse direction (e.g., from [u] to [y]). Here, the peripheral vowels serve as perceptual reference for listeners to discriminate vowels and the listeners show a bias in favoring a “focal” vowel, resulting in asymmetric processing of the vowel pair in different directions. Directional asymmetry was also reported by Kuhl (1991, 1992): listeners’ discrimination from a prototypical to a non-prototypical vowel within a given category is more difficult than the same change in the reverse direction. For instance, listeners were presented with a range of synthesized [i] vowels which varied in F1/F2 and asked to rate the perceived goodness of the vowels. They consistently attached the highest goodness values to vowels within a particular vowel space (Kuhl, 1991). Variants with changes to F1/F2 were synthesized on the basis of the prototype and non-prototype exemplars selected according to the ratings. Compared to a non-prototype exemplar, it is more difficult to discriminate the prototype from its variants (Kuhl, 1992). Therefore, the NLM theory argues that early linguistic experience influences perceptual patterns, such that listeners become biased toward native prototypes. These prototypes in turn function as perceptual magnets for other members within category while stretching the distance between categories (Kuhl, 1992, 1993).

However, neither of the two models are applicable to our study as the difference wave was obtained by subtracting the waveform of the stimulus when presented as standard in one block from that of the same stimulus when presented as deviant in another block. In other words, there is no difference in vowel space or phonetic category between standard and deviant. The MMN component is automatically generated by change-detection and the neurons activated by standards are separate from those activated by deviants (Jacobsen et al., 2003; Näätänen et al., 2005, 2007). The repetition of stimuli, though, might lead to a refractory effect on neurons that are either activated by the standard or the deviant, but not both. Compared to the deviant, the neural response to standards is more likely to be suppressed due to its high probability of occurrence, resulting in a misestimate MMN (Jacobsen and Schröger, 2001; Jacobsen et al., 2003). Adopting physically identical stimuli allows for the generation of genuine MMN responses without contamination by physical differences of the stimuli (Jacobsen and Schröger, 2001; Jacobsen et al., 2003). Note that subtracting the waveform of standard stimuli from that of the deviant one may not completely eliminate the potential influence of N1 on MMN, as the amplitude of N1 elicited by different stimuli varies. Previous studies also found that distinct acoustic properties of segments in a syllable or consonant-vowel transition can lead to potential P1-N1-P2, which may have an effect on the asymmetric activation of MMN and LDN (Martin and Boothroyd, 1999; Miller and Zhang, 2014). Indeed, N1 has been noted as a component which extracts phonological features (cf. Obleser et al., 2004). Future studies could use alternative measurements to separate the effects of MMN and N1, and investigate the influence of the transition within stimuli or vocalic cue on the ERP components (Schröger and Wolff, 1996; Miller and Zhang, 2014).

To sum up, our results provide neurophysiological evidence for the interactive effect of vowels on fricative sibilants in Mandarin. Features such as TONGUE HEIGHT spread on the surface so that unspecified sibilants are influenced by following vowels. When followed by a [HIGH] vowel such as [u], the unspecified sibilant [ʂ] takes on the HEIGHT feature from [u] while the specified [s] retains its own HEIGHT feature [LOW]. Therefore, asymmetries were triggered by the same phonological contrast [su] ∼ [ʂu] in two directions where the surface [HIGH] of the deviant [ʂ] conflicts with the underlying specified [LOW] of the standard [s], while the surface [LOW] activated by [s] does not mismatch with the unspecified [ʂ]. When followed by a [LOW] vowel such as [a], no such asymmetry was observed as there is no conflict between the surface [LOW] from [a] and the underlying specified [LOW]. In addition, the LDN component has demonstrated its reliability in linguistic processing among adults and its deflection pattern is roughly consistent with that of the MMN. Future studies should consider taking this component into consideration when investigating the underspecification of segments in the mental lexicon. In conclusion, not all features are fully specified in the mental lexicon and the specification of a feature such as TONGUE HEIGHT is determined by the number of contrastive segments in a certain language.

## Data Availability Statement

The raw data supporting the conclusions of this article will be made available by the authors, without undue reservation.

## Ethics Statement

The studies involving human participants were reviewed and approved by the Central University Research Ethics Committee, University of Oxford. The patients/participants provided their written informed consent to participate in this study.

## Author Contributions

YM: data collection, formal analysis, methodology, investigation, conceptualization, writing–original draft, and writing–review and editing. SK: methodology, conceptualization, and writing–review and editing. CX: data collection and writing–original draft. HW: writing–review and editing; AL: conceptualization, funding acquisition, methodology, project administration, supervision, writing–original draft, and writing–review and editing. All authors contributed to the article and approved the submitted version.

## Conflict of Interest

The authors declare that the research was conducted in the absence of any commercial or financial relationships that could be construed as a potential conflict of interest.
