# Supplementary material for: Asymmetric Influence of Vocalic Context on Mandarin Sibilants: Evidence From ERP Studies
Source: Front Hum Neurosci. 2021 Apr 22;15:617318. doi: 10.3389/fnhum.2021.617318 (PMC8100247; doi:10.3389/fnhum.2021.617318)
Supplement: Supplementary file 1 [file Table_1.DOCX]

**Supplementary materials**

**Figure 1.** Grand average ERPs to the standard and deviant stimulus waveforms at all selected electrodes for word pair /ʂa/~/sa/.

******

**Figure 2.** Grand-average difference waveforms of /ʂa/_[sa]_ and /sa/_[ʂa]_ conditions at all selected electrodes.

**Figure 3.** Grand average ERPs to the standard and deviant stimulus waveforms at all selected electrodes for word pair /ʂu/~/su/.

**Figure 4.** Grand-average difference waveforms of /ʂu/_[su]_ and /su/_[ʂu]_ conditions at all selected electrodes.
